# Mixed neuroendocrine-non-neuroendocrine neoplasms of the right colon: a case report

**DOI:** 10.11604/pamj.2021.40.243.30627

**Published:** 2021-12-21

**Authors:** Zakaria El Barkaoui, Mohammed Najih, Aboulfeth El Mehdi, Hicham El Majdoubi, Imane El Messaoudi, Mohamed Amine Essaoudi, Mohamed Bouzroud, Sidi Mohammed Bouchentouf, Hakim El Kaoui, Ahmed Bounaim

**Affiliations:** 1 Department of General Surgery, Military Hospital Mohamed V Rabat, Rabat, Morocco,; 2 Department of Pathology, Military Hospital Mohamed V Rabat, Rabat, Morocco

**Keywords:** Ascending colon, neuroendocrine tumor G3, adenocarcinoma, case report

## Abstract

Mixed neuroendocrine-non neuroendocrine neoplasm (MiNENs) is a rare gastrointestinal neoplasm that has been redefined by the World Health Organization (WHO) in 2017 as the association of two types of components, neuroendocrine and non-neuroendocrine, each of them present in at least 30% of the tumour mass. Small case reports and case series have demonstrated the occurrence of this neoplasm in the colon. We here report the case of a 47-year-old man undergoing colonscopy for anemia. This showed impassable polypoidal tumor budding in the right colic flexure. Computerized tomography (CT) scan and magnetic resonance imaging (MRI) showed the presence of liver metastases. As the tumor was hemorrhagic, right hemicolectomy with lymph node dissection was performed. The histological examination showed MiNEN of the ascending colon. The patient received adjuvant chemotherapy.

## Introduction

MiNENs represent a rare diagnosis of the gastro-entero-pancreatic tract [1] that has been redefined by WHO in 2017 as the association of two types of components, a non-neuroendocrine component (most often adenoma/adenocarcinoma) and neuroendocrine tumor (NET G1, NET G2, NEC) [2], each of them must theoretically account for at least 30% of the whole neoplasm [3]. The latest recommendation of the WHO suggests, that each histologic component should be separately graded and evaluated [1,4]. The locations of pathology such as colon are rare [5-7], it´s about 3-9,6% of all colorectal tumors. The right colon is the most common (56%), then the left colon and transverse colon [8]. We present a case of MiNENs of ascending colon.

## Patient and observation

**Patient information**: a 47-year-old man, chronic smoker, with no medical history, presented with progressively worsening pain in the right lower abdominal quadrant in the last 2 months, which was associated with melena, postprandial vomiting, and weight loss of 5 kg for 3 months without anorexia or asthenia.

**Clinical finding**: on examination, he was pale, and on abdominal examination, a palpable mass in the right lower abdominal quadrant that was painful, with dullness on percussion. On rectal examination, the finger pad was covered with blood.

**Figure 1:**
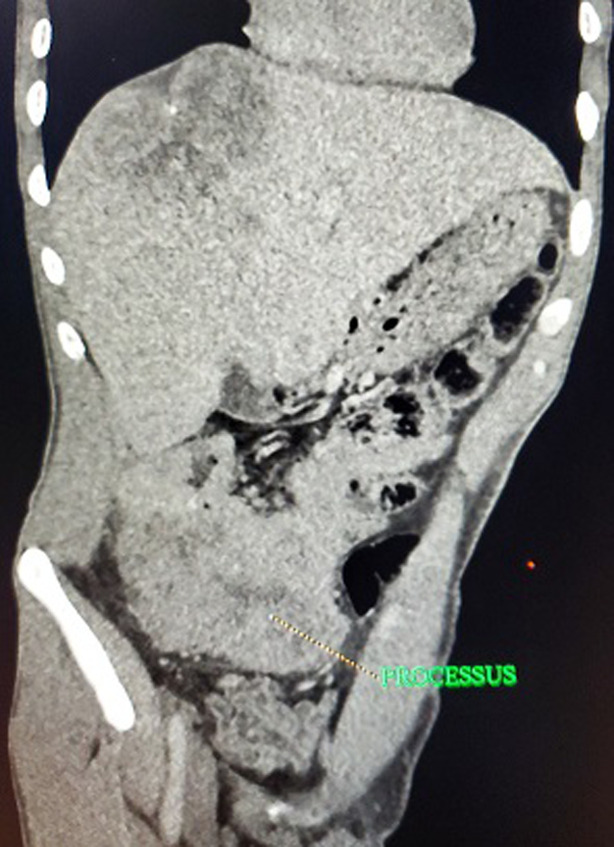
preoperative computed tomography scan, showed a large mass of polylobed appearance of the coecum extended to the colonic angle, associated with hepatic lesion

**Figure 2:**
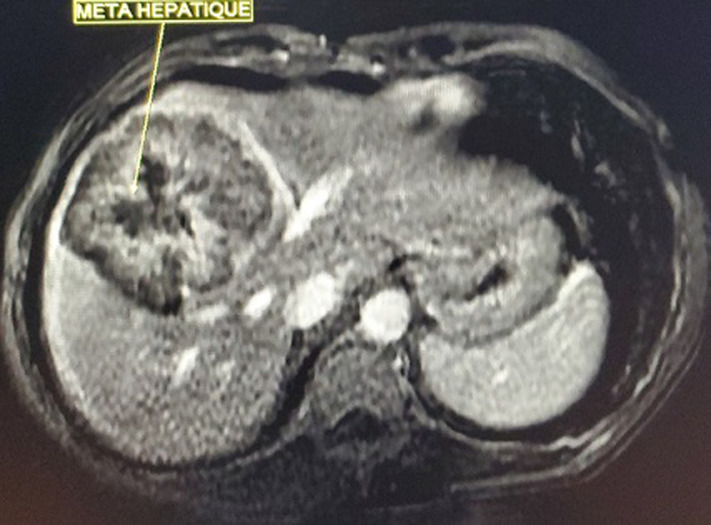
preoperative magnetic resonance imaging of the liver showing hepatic lesions with central necrosis

**Diagnostic assessment**: laboratory data showed microcytic hypochromic anemia with; hemoglobin (Hg) at 6.5g/dl, Mean Blood Volume (MBV) at 58.2 fl, and Mean Corpuscular Hemoglobin Content (MCHC) at 17.4 pg. Serum levels of tumor markers: Carcinoembryonic antigen (CEA) and carbohydrate antigen 19-9 (CA 19-9) were normal. Colonoscopy for anemia examination showed an impassable polypoidal budding formation of the right colonic angle. The biopsy is in favor of a well-differentiated and invasive adenocarcinoma on a degenerated tubular villous polyp. The Computed tomography (CT) scan was performed, showed a large mass of polylobed appearance of the coecum extended to the colonic angle measuring 115x67x132mm (Figure 1) preoperative computed tomography (CT) scan, showed a large mass of polylobed appearance of the coecum extended to the colonic angle, associated with hepatic lesion), associated with hepatic lesions, which the most voluminous are located at the level of the hepatic dome measuring 81x90mm and segment VI measuring 72x60mm confirmed by magnetic resonance imaging (MRI) (Figure 2) preoperative computed tomography (CT) scan, showed a large mass of polylobed appearance of the coecum extended to the colonic angle, associated with hepatic lesion). A liver biopsy was in favor of a well differentiated adenocarcinoma.

**Therapeutic intervention**: because the tumor was hemorrhagic, it was decided to perform a right hemicolectomy with lymphadenectomy and ileo-transverse anastomosis. The postoperative evolution was good, the patient was discharged on the 8^th^ postoperative day.

**Figure 3:**
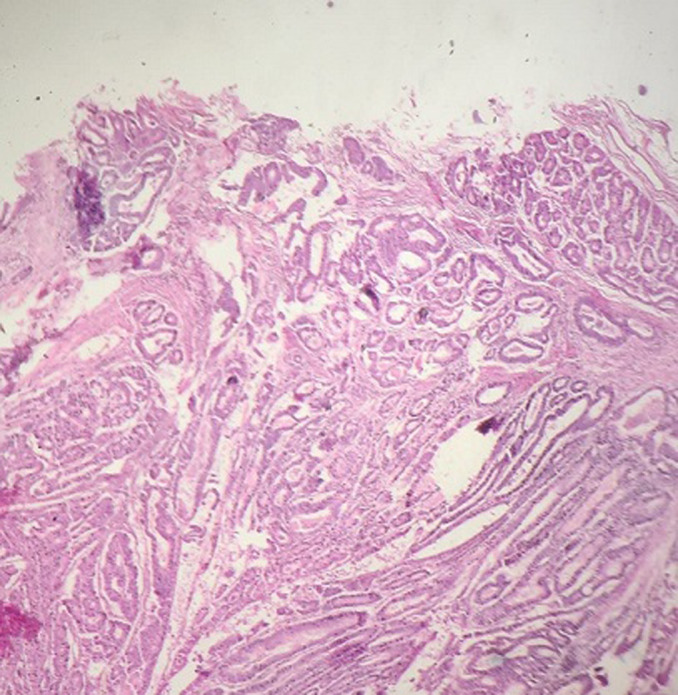
adenocarcinomatous component

**Figure 4:**
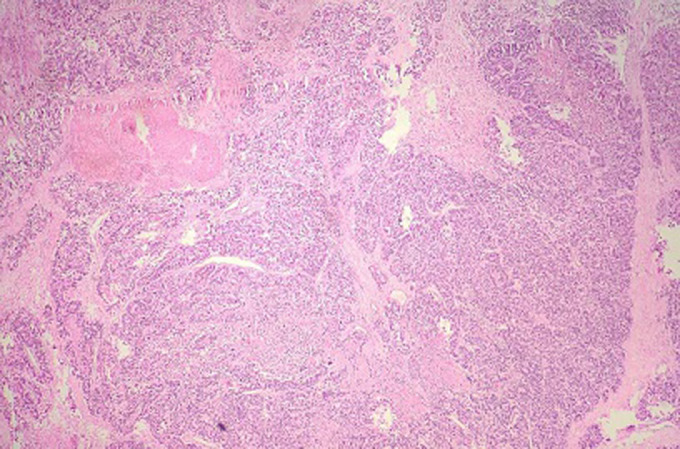
neuroendocrine component

**Figure 5:**
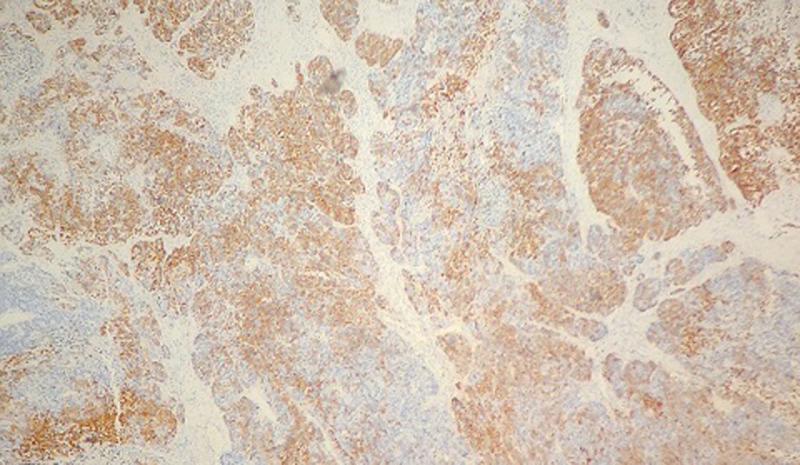
immunohistochemical study, positive labeling of neuroendocrine tumor cells by synaptophysin

**Figure 6:**
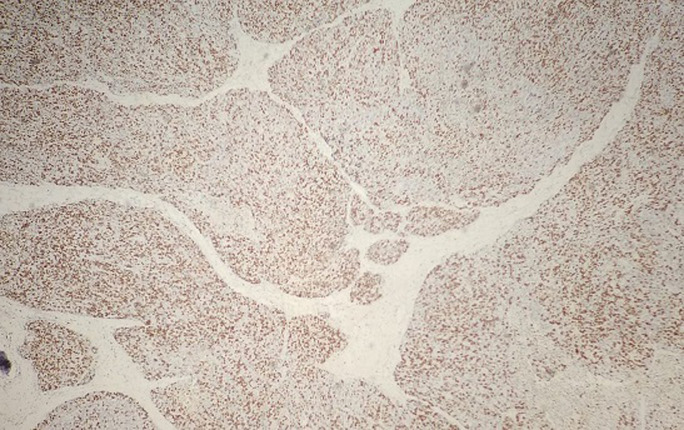
tumor cells were positive for Ki-67 and estimated at 60%

**Pathological finding**: on the histological examination, the tumor was 20x15x16cm in size. it had a 100% circumferential ulcerative-bourgeons appearance. Microscopic examination showed a proliferating colonic mucosa made of clumps, the presence of large foci of tumor necrosis. Mitoses were estimated at 43 mitoses/10 field. He also showed an adenocarcinomatous contingent (Figure 3) preoperative computed tomography (CT) scan, showed a large mass of polylobed appearance of the coecum extended to the colonic angle, associated with hepatic lesion) estimated at 15% of the tumor surface made of tubes of cribriform masses bordered by obviously atypical cells. The cytoplasm was abundantly eosinophilic. The tumor infiltrated all tunics and perforated the colonic wall, with the presence of vascular embolism and absence of perineural sheaths. Eight Of the 29 lymph nodes removed were invaded by the neuroendocrine contingent (Figure 4) preoperative computed tomography (CT) scan, showed a large mass of polylobed appearance of the coecum extended to the colonic angle, associated with hepatic lesion) without capsular erosion. The immunohistochemical study showed; synaptophysin: diffuse expression in the neuroendocrine contingent (Figure 5) preoperative computed tomography (CT) scan, showed a large mass of polylobed appearance of the coecum extended to the colonic angle, associated with hepatic lesion). CD56 and chromogranin: were negative. Ki67 was estimated at 60% (Figure 6) preoperative computed tomography (CT) scan, showed a large mass of polylobed appearance of the coecum extended to the colonic angle, associated with hepatic lesion). Based on these pathological findings, the tumor was diagnosed as mixed adeno-neuroendocrine carcinoma (MiNEN), associating a majority G3 neuroendocrine tumor (85%) and a moderately differentiated adenocarcinoma contingent (15%). As this was metastatic cancer and the patient is young, postoperative adjuvant chemotherapy was performed.

**Consent**: written informed consent was obtained from the patient for publication of this case report and any accompanying images. A copy of the written consent is available for review by the Editor of this journal

## Discussion

Colonic MiNENs are one of the most common types of MiNENs. Pure NETs and MiNENs represent 1.1 and 2.4% of surgically resected colorectal neoplasms, respectively [9], while colonic MiNENs constitute 14-20% of colonic neuroendocrine neoplasms (NEN) [10-11]. Most colonic neuroendocrine neoplasms (NENs) (85%) are poorly differentiated neuroendocrine carcinomas (PDNECs), containing a non-neuroendocrine component in 25-40% of cases [12-16], which is generally an adenocarcinoma (45-64%), but adenoma or squamous cell carcinoma is found in about 30-35% and 5% of cases, respectively, derived mostly from the mucosa, while the neuroendocrine component frequently develops from the deepest layer of the colon wall and may be missed when no further resection of the primary tumor is done after biopsies [17].

The prognosis of colorectal MiNEN is actually worse than that of pure adenocarcinoma, and most similar to that of PDNEC, especially at the metastatic stage [10,12]. The metastatic risk is considered to be correlated with the grade of the neuroendocrine component [14]. Indeed, when the neuroendocrine component is PDNEC, it is practically always present in metastasis, compared to adenocarcinoma which is present in only about 30% of cases [14]. La Rosa *et al*. classified MiNEN into three grades (high, intermediate, and low) according to the prognosis and found that the grade was useful for determining treatment strategies [18]. Low-grade MiNEN is a combination of well-differentiated NET and adenoma, which is rare and has been reported primarily in the gastrointestinal tract [19]. NETs can metastasize and need to be treated as pure NETs. Intermediate grade MiNEN is a combination of well-differentiated NET and non-neuroendocrine cancer. Prognosis is usually determined by the non-neuroendocrine carcinoma, but neuroendocrine components must be considered as prognostic factors, particularly when NET G3 components are implicated [19]. If it is resectable, surgery should be done for therapeutic purposes, and if metastases are involved, the components found in the metastases have to be targeted with chemotherapy or drugs active against both components. High-grade MiNEN are the most frequent cases, combining NEC with adenocarcinoma or adenoma, with NEC generally considered the most aggressive component [10,12,19-24]. If the lesion is localized without distant metastases, it can be removed surgically with perioperative chemotherapy. Systemic chemotherapy is recommended in situations involving distant metastases.

In our case, the adenocarcinoma segment represented 15% of the total tumor tissue, infiltrating all tunics with perforation of the colonic wall, while the neuroendocrine component represented 85% of the total tumor tissue and developed around the colonic mucosa, so this case was diagnosed as a combined/biphasic type of MiNEN. The current tumor was histologically composed of G3 NET and adenocarcinoma, with the presence of distant metastases and classified as High-grade MiNEN according to the classification of Rosa *et al*. Adjuvant chemotherapy was considered, as a resection was already obtained since the tumor was symptomatic.

## Conclusion

MiNEN of ascending colon is very rare. Although our case was a high-grade lesion combining NEC with adenocarcinoma, which is considered the most aggressive component and the prognosis is expected to be bad. The treatment management should be based on the most aggressive component of neoplasia, which can be determined reliably only by analysis of a resected specimen, even if it is less than 30% of the neoplasm.
